# Enhanced expression of natural cytotoxicity receptors on cytokine-induced memory-like natural killer cells correlates with effector function

**DOI:** 10.3389/fimmu.2023.1256404

**Published:** 2023-10-16

**Authors:** Sofía Carreira-Santos, Nelson López-Sejas, Marina González-Sánchez, Eva Sánchez-Hernández, Alejandra Pera, Fakhri Hassouneh, Esther Durán, Rafael Solana, Javier G. Casado, Raquel Tarazona

**Affiliations:** ^1^ Immunology Unit, Department of Physiology, Universidad de Extremadura, Cáceres, Spain; ^2^ Immunology and Allergy Group (GC01), Maimonides Biomedical Research Institute of Córdoba (IMIBIC), Córdoba, Spain; ^3^ Department of Cell Biology, Physiology and Immunology, Universidad de Córdoba, Córdoba, Spain; ^4^ Anatomy and Comparative Pathological Anatomy Unit, Department of Animal Medicine, Faculty of Veterinary Medicine, Universidad de Extremadura, Cáceres, Spain; ^5^ Immunology and Allergy Service, Reina Sofia University Hospital, Cordoba, Spain; ^6^ Centro de Investigación Biomédica En Red (CIBER) de Enfermedades Cardiovasculares, Instituto de Salud Carlos III (ISCIII), Madrid, Spain; ^7^ RICORS-TERAV Network, Instituto de Salud Carlos III (ISCIII), Madrid, Spain; ^8^ Institute of Molecular Pathology Biomarkers, Universidad de Extremadura, Cáceres, Spain

**Keywords:** NK cells, memory-like, cytokine-induced memory-like NK cells, NKG2D, natural cytotoxicity receptors, degranulation capacity, cancer immunotherapy

## Abstract

**Introduction:**

Natural killer (NK) cells are a key component of the innate immune system, involved in defending the host against virus-infected cells and tumor immunosurveillance. Under *in vitro* culture conditions, IL-12/15/18 can induce a memory-like phenotype in NK cells. These cytokine-induced memory-like (CIML) NK cells possess desirable characteristics for immunotherapies, including a longer lifespan and increased cytotoxicity.

**Methods:**

In this study, NK cells were isolated from peripheral blood of healthy donors and stimulated with IL-12/15/18 to induce a memory-like phenotype or with IL-15 alone as a control. After seven days of culture, multiparametric flow cytometry analysis was performed to evaluate the phenotypic and functional profiles of CIML and control NK cells.

**Results:**

Our results showed a significantly higher expression of CD25, CD69, NKG2D, NKp30, NKp44, NKp46, TACTILE, and Granzyme B in CIML NK cells compared to control NK cells. In contrast, KIR2D expression was significantly lower in CIML NK cells than in control NK cells. Moreover, functional experiments demonstrated that CIML NK cells displayed enhanced degranulation capacity and increased intracellular IFN-γ production against the target cell line K562. Interestingly, the degranulation capacity of CIML NK cells was positively correlated with the expression of the activating receptors NKp46 and NKp30, as well as with the inhibitory receptor TACTILE.

**Discussion:**

In conclusion, this study provides a deep phenotypic characterization of *in vitro*-expanded CIML NK cells. Moreover, the correlations found between NK cell receptors and degranulation capacity of CIML NK cells allowed the identification of several biomarkers that could be useful in clinical settings.

## 1 Introduction

Natural Killer (NK) cells are innate lymphocytes that are essential not only in host defense against virus-infected cells, but also in tumor immune surveillance (1, 2). The effector function of NK cells is regulated through a rigorous balance of signals that are mediated by the interaction between the activating and inhibitory receptors expressed on the surface of NK cells and their corresponding ligands on target cells (2–4).

The interaction between NK activating receptors, including NKG2D, NKG2C, DNAM-1, NCRs (NKp46, NKp44, and NKp30), and NKp80, with their corresponding ligands on tumor cells results in the activation of NK cells, leading to enhanced NK cell cytotoxicity (3–5). The ligands for these receptors, MICA/B (ligands for NKG2D), HLA-E (ligand for NKG2C), CD122, CD155 (both ligands for DNAM-1) (3–5), AICL (ligand for NKp80) (6, 7), and B7-H6 (ligand for NKp30) (8, 9), are frequently upregulated in tumor cells and can even be entirely absent in healthy cells, as is the case for B7-H6. In addition, the CD16 molecule is a low-affinity Fc receptor for IgG, enabling NK cell activation and triggering antibody-dependent cellular cytotoxicity (ADCC) without requiring additional receptor signals (10, 11).

It is interesting to note that CD112 and CD155 also serve as ligands for NK inhibitory receptors, such as TIGIT (which recognizes both CD112 and CD155) and TACTILE (which recognizes CD155). The interaction between these ligands and NK inhibitory receptors leads to a decrease in the cytotoxic capacity of NK cells while promoting tumor cell invasion and migration (12). More recently, several inhibitory receptors have been described in NK cells: LAG-3 (lymphocyte-3 activation gene), which binds to LSECtin (liver and lymph node sinusoidal endothelial cell C-type lectin) on tumor cells, TIM-3 (mucin domain-containing protein 3), which binds to multiple ligands such as galectin-9, HMGB1, Ceacam-1 and Phosphatidylserine, and PD-1, which binds to PD-L1 (programmed death-ligand) (3).

Traditionally, NK cells have been characterized as innate immune cells due to their rapid proliferation and effector function, including cytotoxicity, and cytokine production (IFN-γ and TNF-α), without prior sensitization or antigen specificity (13–16). However, recent studies have revealed that NK cells can undergo clonal expansion and give rise to long-lived memory-like NK cells which exhibit rapid degranulation and cytokine production upon reactivation (15, 16). The development of this memory-like phenotype in NK cells can occur through three distinct scenarios: hapten-specific, virus-specific, and cytokine-induced (16–18).

Cytokine-induced memory-like (CIML) NK cells are a subset of NK cells that after a short *in vitro* stimulation with IL-12, IL-15, and IL-18, exhibit enhanced functional and phenotypic characteristics (14, 18, 19). Despite being antigen nonspecific, CIML NK cells retain memory of previous activation and persist within the host for long periods of time, exhibiting an enhanced functional and proliferative capacity (13, 19).

Their longer half-life, together with the maintenance of their highly cytotoxic and anti-tumor responses in the immunosuppressive tumor microenvironment, makes CIML NK cells a great candidate for future immunotherapies (13, 20, 21). In fact, multiple preclinical and clinical studies have demonstrated the therapeutic potential of CIML NK cells in various cancer models, including ovarian cancer, melanoma, acute myeloid leukemia (AML), and lymphoma (22–25). All these promising results, together with their functional and phenotypic characteristics, support the idea that CIML NK cells may have a key role in combating diverse types of malignancies.

In this study, we conducted a comprehensive analysis of the phenotype and functional capacity of *in vitro-*expanded CIML NK cells. Our findings revealed that, after 7 days of culture, CIML NK cells exhibited a significantly higher expression of CD25, CD69, NKG2D, natural cytotoxicity receptors (NCRs), and Granzyme B compared to control NK cells. Additionally, CIML NK cells showed significantly higher percentages of expression of the inhibitory receptors NKG2A and TACTILE (CD96), along with significantly lower expression of KIR2D. Furthermore, we found that CIML NK cells exhibited a greater degranulation capacity and higher production of IFN-γ against the target cell line K562 than control NK cells. Moreover, to further explore the relationship between the phenotype and functionality of CIML NK cells, we conducted a multiple correlation analysis that revealed positive correlations between the degranulation capacity of CIML NK cells and the expression of activating receptors (NKp46, and NKp30), and inhibitory receptors (TACTILE). Here we suggest that the phenotypic analysis of these molecules prior to adoptive cell therapy may help to predict the functionality and therapeutic potential of CIML NK cells.

## 2 Materials and methods

### 2.1 Sample processing and NK cell culture

NK cells were isolated from the buffy coats of healthy donors (n = 8) by a negative selection process using the NK cell enrichment cocktail RosetteSep™ (STEMCELL Technologies, Vancouver, BC, Canada) and density gradient centrifugation on Lymphoprep™ (STEMCELL Technologies). The cells were then cultured in HyClone RPMI-1640 (Cytiva, Marlborough, MA, USA) supplemented with 1% Sodium Pyruvate, 1% GlutaMAX™ (Gibco™, ThermoFisher Scientific, Waltham, MA, USA), 1% Penicillin-Streptomycin (Lonza Ltd., Verviers, Belgium), and 10% Human Serum AB male (Biowest, Nuaillé, France).

To generate CIML NK cells, the cells were seeded in 24 well plates at a density of 1x10^6^ cells/mL and stimulated overnight (~16h) with 10 ng/mL rhIL-12, 1 ng/mL rhIL-15 (PrepoTech, Rocky Hill, NJ, USA), and 50 ng/mL rhIL-18 (MBL International, Woburn, MA, USA). In parallel, NK cells incubated overnight with 1 ng/mL rhIL-15 were defined as control NK cells. After the initial stimulation, the cells were washed twice and seeded again in the presence of 1 ng/mL rhIL-15. Cell culture medium supplemented with rhIL-15 was replaced on day 4.

The ethics committee of the University of Extremadura (Ref.: 118//2020) approved the study, and informed consent was obtained following the Declaration of Helsinki.

### 2.2 Cell lines

The human erythroleukemic cell line K562 and human melanoma cell line MaMel56 (from OISTER and ESTDAB projects) were cultured in HyClone RPMI-1640 supplemented with 10% Foetal Bovine Serum (Gibco™, ThermoFisher Scientific, Waltham, MA, USA), 1% GlutaMAX™, and 1% Penicillin-Streptomycin. A non-enzymatic cell dissociation solution (Sigma-Aldrich, St. Louis, MO, USA) was used to detach the MaMel56 cells from the culture flask.

### 2.3 Flow cytometry analysis

The phenotypic and functional profiles of both the CIML and control NK cells were analyzed by multiparametric flow cytometry using a panel of commercially available antibodies (**Supplementary Tables 1**-**3**). The cells were stained (**Supplementary Table 1**) with fluorochrome-conjugated antibodies and incubated for 30 min at room temperature. For intracellular staining, the cells were fixed and permeabilized with the IntraCell kit from Immunostep (Salamanca, Spain) following the manufacturer’s instructions; intracellular antibodies (**Supplementary Table 2**) were incubated for 30 min at room temperature. The cells were washed and resuspended in PBS before flow cytometry analysis. Isotype-matched antibodies and fluorescence minus one (FMO) were used as controls to ensure proper gating.

Briefly, after doublet exclusion, lymphocytes were gated according to their size and granularity using forward (FSC) and side scatter (SSC) detectors, and NK cells were identified within the lymphocyte gate as CD3− CD56+. Individual gates were defined for antibodies included in the panel for CD3−CD56+ cells. A detailed outline of the gating strategy is provided in the **Supplementary Material** (**Supplementary Figure 1**).

Flow cytometry was performed using MACSQuant Analyzer 10 (Miltenyi Biotec, Bergisch Gladbach, Germany) and analyzed using FlowLogic v.8.6. (Inivai Technologies, Mentone, Victoria, Australia).

### 2.4 NK cell degranulation and cytokine production assays

The functionality of CIML and control NK cells was determined as specific degranulation capacity after 7 days of culture. Degranulation capacity was estimated by CD107a and CD107b expression on NK cells, and specific degranulation capacity of NK cells against target cells was calculated after subtracting the basal degranulation in the absence of target cell from the degranulation in the presence of target cell. Briefly, CIML and control NK cells (5x10^5^ cells) were stimulated with the target cells, the K562 and MaMel56 cell lines, at a 1:1 effector:target (E:T) ratio. In order to favor cell-to-cell contact, effector and target cells were transferred into sterile 5 mL polystyrene round-bottom tubes (Falcon^®^, Corning Inc., Corning, NY, USA) and centrifuged for 5 min at 200*g*. Then, cells were resuspended in 200 µL of cell culture medium and stained with the FITC-conjugated anti-CD107a (H4A3; BD Biosciences, San José, CA, USA) and FITC-conjugated anti-CD107b (H4B4; BD Biosciences) monoclonal antibodies. Thereafter, the protein transport inhibitors GolgiStop™ and GolgiPlug™ (BD Biosciences), were added to each tube following the manufacturer’s indications, and the cells were incubated at 37°C, 5% CO_2_, and 95% humidity for 6 hours. The cells were then stained with the appropriate antibodies (**Supplementary Table 3**) following the method explained above and analyzed by flow cytometry.

The assessment of intracellular cytokine production (IFN-γ and TNF-α) was conducted on a different group of donors (n = 7). For this purpose, specific cytokine production of NK cells against target cells was calculated after subtracting the basal production in the absence of target cell. CIML and control NK cells were stimulated as previously described for degranulation assays, in the presence of protein transport inhibitors GolgiStop™ and GolgiPlug™. Finally, after a 6h incubation, cells were stained with the appropriate and intracellular antibodies (**Supplementary Table 3**), as described in section 2.3, and analyzed by flow cytometry.

### 2.5 Statistical analysis and graphical representation

Prior to the statistical analysis, the normality of the datasets was checked using the Shapiro-Wilk normality test. The non-parametric Friedman test, followed by pairwise comparisons (Durbin-Conover test), was used for multiple comparison analysis, while the non-parametric Wilcoxon signed-rank test was used to study paired samples. The statistical analysis of the samples was carried out in the open-source statistical software Jamovi v2.3.21 (Sydney, Australia). The correlation analysis between the different biomarkers was based on the Pearson correlation coefficient and conducted on R-Studio v2022.12.0 + 353.

The graphical representations were performed using GraphPad Prism v8.0.1 (San Diego, CA, USA), except for the correlation matrix, which was obtained through R-Studio.

Simplified Presentation of Incredibly Complex Evaluations (SPICE) software was used for pie chart representation and comparison. The statistical comparison of the distributions was accomplished by a non-parametric partial permutation test (26).

## 3 Results

### 3.1 *In vitro* expansion of CIML NK cells can be achieved with IL-12/15/18 cytokines

In our experimental conditions, NK cells were firstly enriched from peripheral blood lymphocytes using RosetteSep™. In all cases, NK cell purity (defined as CD3− CD56+ cells) resulted in a mean purity of 76.10% ± 9.66%. After 7 days of culture, CIML NK cells and control NK cells reached a purity of 82.26% ± 11.21% and 80.62% ± 12.78% respectively. Furthermore, while there were no statistically significant differences in the proliferation rates and absolute cell counts between CIML and control NK cells, it is worth noting that in 6 out of the 8 donors included in this study, CIML NK cells displayed higher absolute cell counts compared to control NK cells after 7 days of culture (**Supplementary Figure 2**).

In addition, the assessment of NK cell viability during the experimental phase indicated that on day 1, the viability of both CIML and control NK cells fell within the range of 85% to 90%. Subsequently, after 7 days of culture, a notable increase in cell viability was observed, with values ranging from 95% to 99% for both CIML and control NK cells.

### 3.2 The phenotypic profiles of CIML and control NK cells are significantly different

In order to study the changes in activating and inhibitory NK cell receptors, and the effect of stimulation with IL-12/15/18 on NK cells, the phenotype of both CIML and control NK cells was studied at different time points: right after the reception and processing of the samples (D0), 16 h after the initial stimulation with cytokines (D1), and after a week of expansion and culture with 1 ng/mL rhIL-15 (D7). The results presented herein correspond to those obtained at D7; for a more comprehensive understanding of the analyses conducted at D0 and D1, **Supplementary Material** has been provided (**Supplementary Figures 3**-**6**).

**Figure 1:**
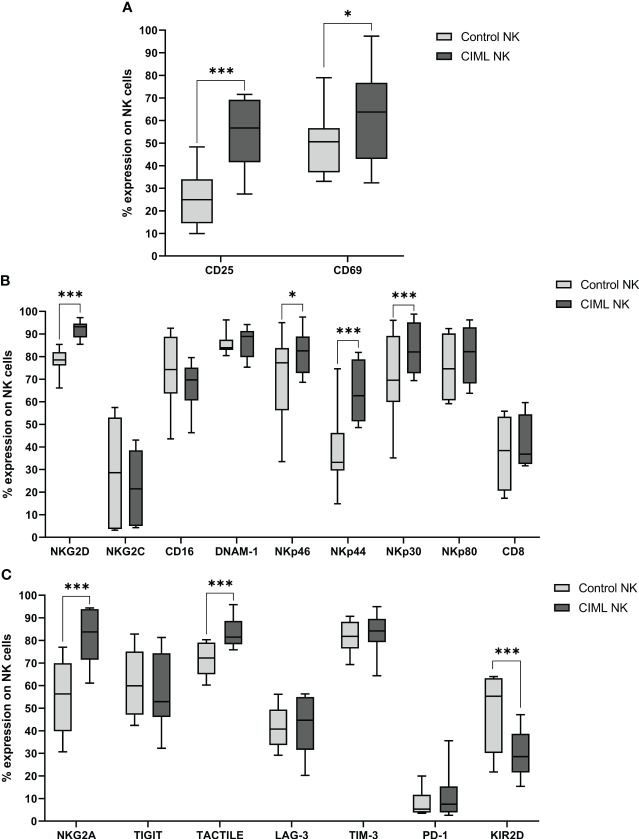
Phenotypic analysis of CIML and control NK cells after 7 days of culture. **(A)** Expression of the activating markers CD25 and CD69. **(B)** Expression of NK cell activating receptors and CD8. **(C)** Expression of NK cell inhibitory receptors. *p*-values were calculated by using the non-parametric Friedman test, followed by pairwise comparisons (Durbin-Conover test), * *p* ≤ 0,05, *** *p* ≤ 0,001.

After 7 days of culture, expression of NK cell activation markers CD25 (*p* < 0.001) and CD69 (*p* = 0.044) was significantly higher in CIML NK cells than in control NK cells (**Figure 1A**). Furthermore, the phenotypic profile of activating NK cell receptors was also studied at D7. In this analysis, NKG2D, NKp46, NKp44, and NKp30 receptors were significantly increased on CIML NK cells when compared to control NK cells (*p* < 0.001 for NKG2D, NKp44, and NKp30; *p* = 0.011 for NKp46). No statistical significance was observed in the expression of NKG2C, CD16, DNAM-1, NKp80, and CD8 (**Figure 1B**).

Regarding inhibitory receptors, CIML NK cells expressed significantly higher percentages of NKG2A and TACTILE (*p* < 0.001, respectively), and significantly lower levels of KIR2D (*p* < 0.001) than control NK cells after 7 days of culture. No significant differences were observed in TIGIT, LAG-3, TIM-3, and PD-1 (**Figure 1C**).

**Figure 2:**
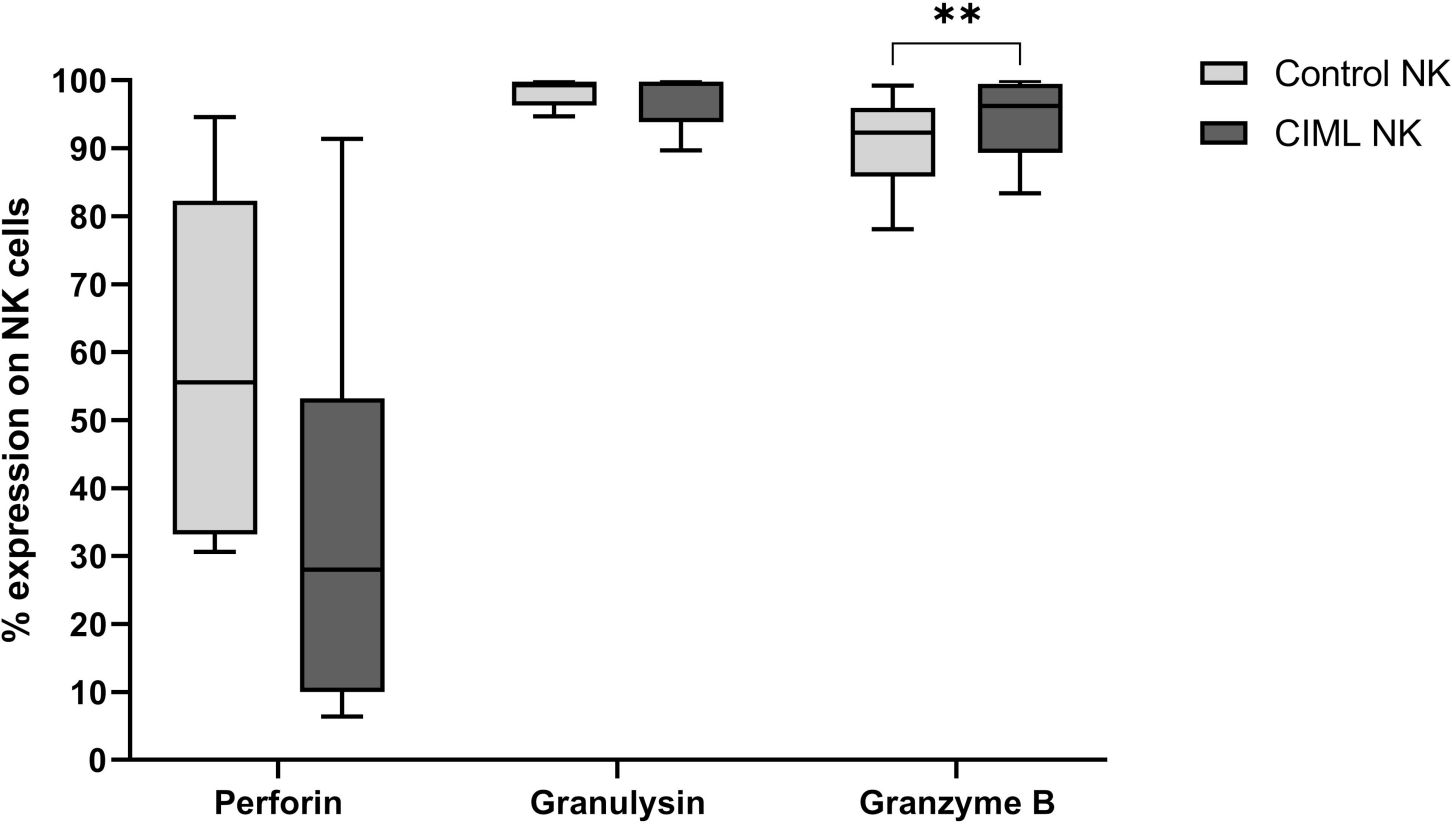
Expression of the cytotoxic proteins Perforin, Granulysin, and Granzyme B in CIML and control NK cells after 7 days of culture. *p*-values were calculated by using the non-parametric Friedman test, followed by pairwise comparisons (Durbin-Conover test), ** *p* ≤ 0,01.

Among the cytotoxic proteins, after 7 days of culture, CIML NK cells showed a significantly higher expression of Granzyme B than control NK cells (*p* = 0.005). No significant differences were observed in Perforin and Granulysin (**Figure 2**).

### 3.3 Phenotypic profiling and subset analysis of CIML and control NK cells based on the co-expression of activating and inhibitory receptors

To further analyze the phenotypic profile of both CIML and control NK cells, and to perform a deep analysis of different NK cell subsets, the co-expression of activating and inhibitory receptors was determined using FlowLogic’s Boolean gating and graphically represented with the SPICE 6.1 software.

**Figure 3:**
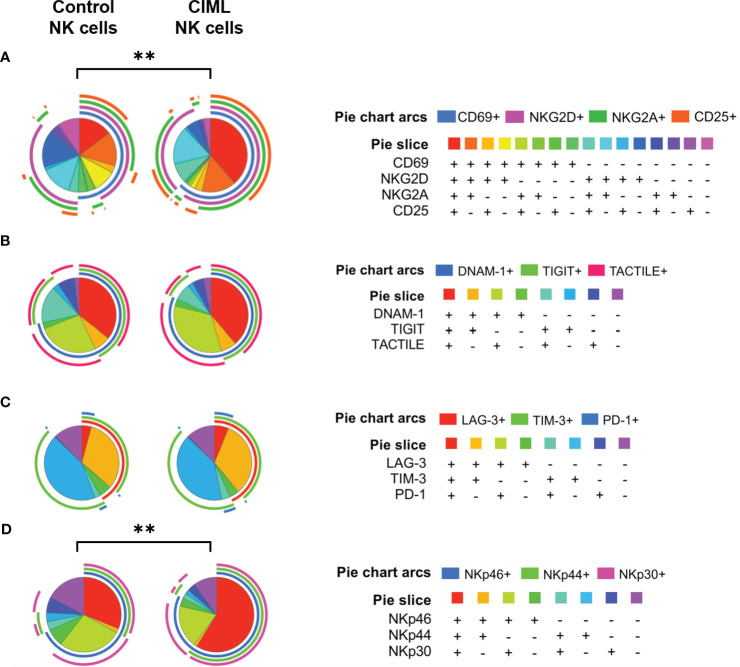
Co-expression of activating and inhibitory receptors in CIML and control NK cells after 7 days of culture. **(A)** Pie charts represent the percentages of CIML and control NK cells expressing CD69, NKG2D, NKG2A, and CD25. **(B)** Pie charts represent percentages of CIML and control NK cells expressing DNAM-1, TIGIT, and TACTILE. **(C)** Pie charts represent percentages of CIML and control NK cells expressing LAG-3, TIM-3, and PD-1. **(D)** Pie charts represent percentages of CIML and control NK cells expressing NKp46, NKp44, and NKp30. *p*-values were calculated by a non-parametric partial permutation test, ** *p* ≤ 0,01.

Sixteen different combinations were analyzed for the following markers: CD69, NKG2D, NKG2A, and CD25. The in-depth phenotypic profiling of CIML and control NK cells revealed statistically significant differences (*p* = 0.0023) in the distribution of NK cell subsets between CIML and control NK cells (**Figure 3A**). Eight different combinations were analyzed for DNAM-1, TIGIT, and TACTILE (**Figure 3B**), as well as for LAG-3, TIM-3, and PD-1 (**Figure 3C**), and NKp46, NKp44, and NKp30 (**Figure 3D**). The distribution of NK cell subsets according to NCRs was significantly different when comparing CIML and control NK cells (*p* = 0.0049).

**Figure 4:**
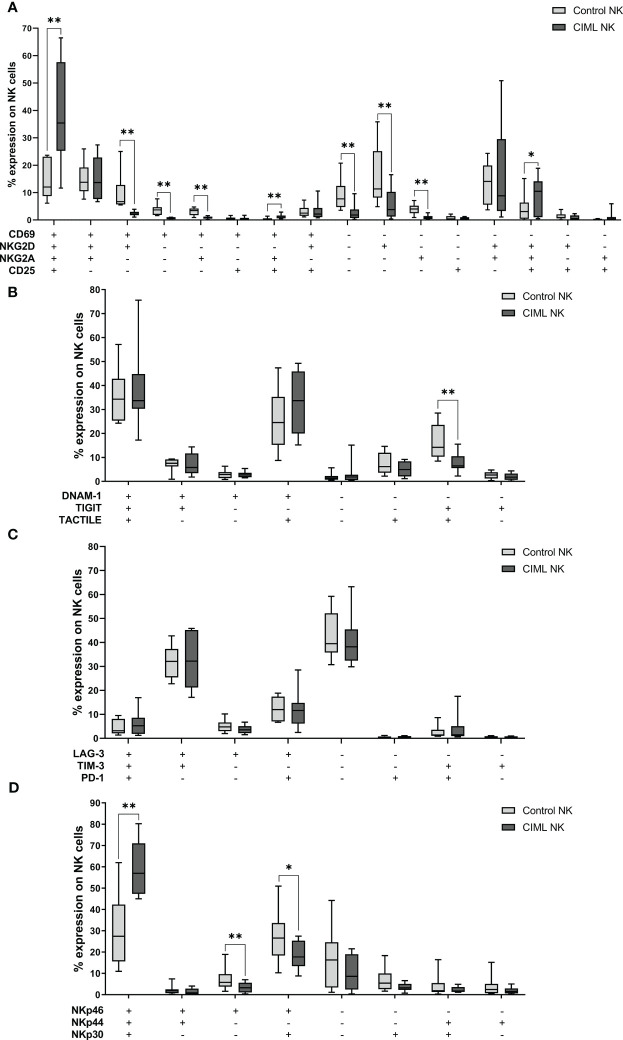
Co-expression of activating and inhibitory receptors in CIML and control NK cells after 7 days of culture. **(A)** Expression of CD69, NKG2D, NKG2A, and/or CD25. **(B)** Expression of DNAM-1, TIGIT, and/or TACTILE. **(C)** Expression of LAG-3, TIM-3, and/or PD-1. **(D)** Expression of NKp46, NKp44, and/or NKp30. *p*-values were calculated by pairwise comparison using the non-parametric Wilcoxon test, * *p* ≤ 0,05, ** *p* ≤ 0,01.

In order to identify differences between the different subsets in CIML and control NK cells, each combination of markers was further analyzed separately. The statistical analysis demonstrated that when compared to control NK cells, CIML NK cells had significantly higher percentages of the CD69+NKG2D+NKG2A+CD25+ subset (*p* = 0.008) than control NK cells. Control NK cells, however, showed significantly higher levels of the CD69−NKG2D−NKG2A−CD25− subset (*p* = 0.008) than CIML NK cells (**Figure 4A**). For a more comprehensive understanding of the evolution of these combinations of markers at the different times of culture (from D0 to D7), refer to the **Supplementary Material** (**Supplementary Figure 7**).

Moreover, after 7 days of culture, control NK cells showed significantly higher percentages of the DNAM-1−TIGIT+TACTILE+ subset (*p* = 0.008) than CIML NK cells (**Figure 4B**). On another note, there were no significant differences between the different combinations of LAG-3, TIM-3, and PD-1 (**Figure 4C**). As for the NCRs, significantly higher percentages of the NKp46+NKp44+NKp30+ subset (*p* = 0.008) were detected for CIML NK cells when compared to CIML NK cells, while the percentages of the NKp46+NKp44−NKp30− (*p* = 0.008) and NKp46+NKp44−NKp30+ (*p* = 0.039) subsets were significantly higher in control NK cells than in CIML NK cells (**Figure 4D**).

### 3.4 CIML NK cells showed higher degranulation and intracellular IFN-γ production against the target cell line K562 than control NK cells

**Figure 5:**
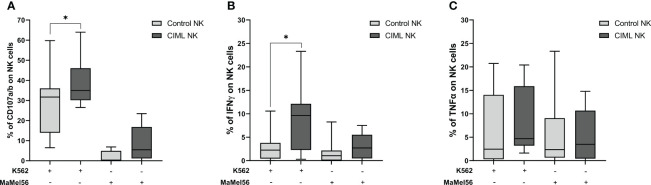
Specific degranulation capacity and production of intracellular IFN-γ and TNF-α in CIML and control NK cells after 7 days of culture. Specific degranulation capacity of NK cells was calculated after subtracting the basal degranulation in the absence of target cell from the degranulation in the presence of target cell. **(A)** Degranulation capacity of CIML and control NK cells against K562 and MaMel56. **(B)** Intracellular IFN-γ production of CIML and control NK cells against K562 and MaMel56. **(C)** Intracellular TNF-α production of CIML and control NK cells against K562 and MaMel56. *p*-values were calculated by pairwise comparison using the non-parametric Wilcoxon test, * *p* ≤ 0,05.

Determination of NK cell degranulation is frequently used as an indirect measure of NK cell cytotoxic activity (27). In order to evaluate the functionality of CIML and control NK cells, their degranulation capacity was tested *in vitro* using K562 and MaMel56 cell lines as targets (E:T = 1:1). Our results demonstrated that CIML NK cells showed a significantly higher degranulation capacity against K562 cell line than control NK cells (*p* = 0.031). The degranulation capacity of both CIML and control NK cells, however, was very low when targeting the melanoma cell line MaMel56 and did not show any statistical differences (**Figure 5A**).

In terms of intracellular cytokine production, our results indicated a significant increase in IFN-γ production by CIML NK cells against the K562 cell line compared to control NK cells (p = 0.027). However, no statistically significant differences were found in IFN-γ production between CIML and control NK cells against the melanoma cell line MaMel56 (**Figure 5B**). Additionally, there were no statistically significant differences in TNF-α production between CIML and control NK cells (**Figure 5C**).

### 3.5 Multiple correlation analysis revealed positive correlations between the degranulation capacity of NK cells and the expression of activating and inhibitory receptors

**Figure 6:**
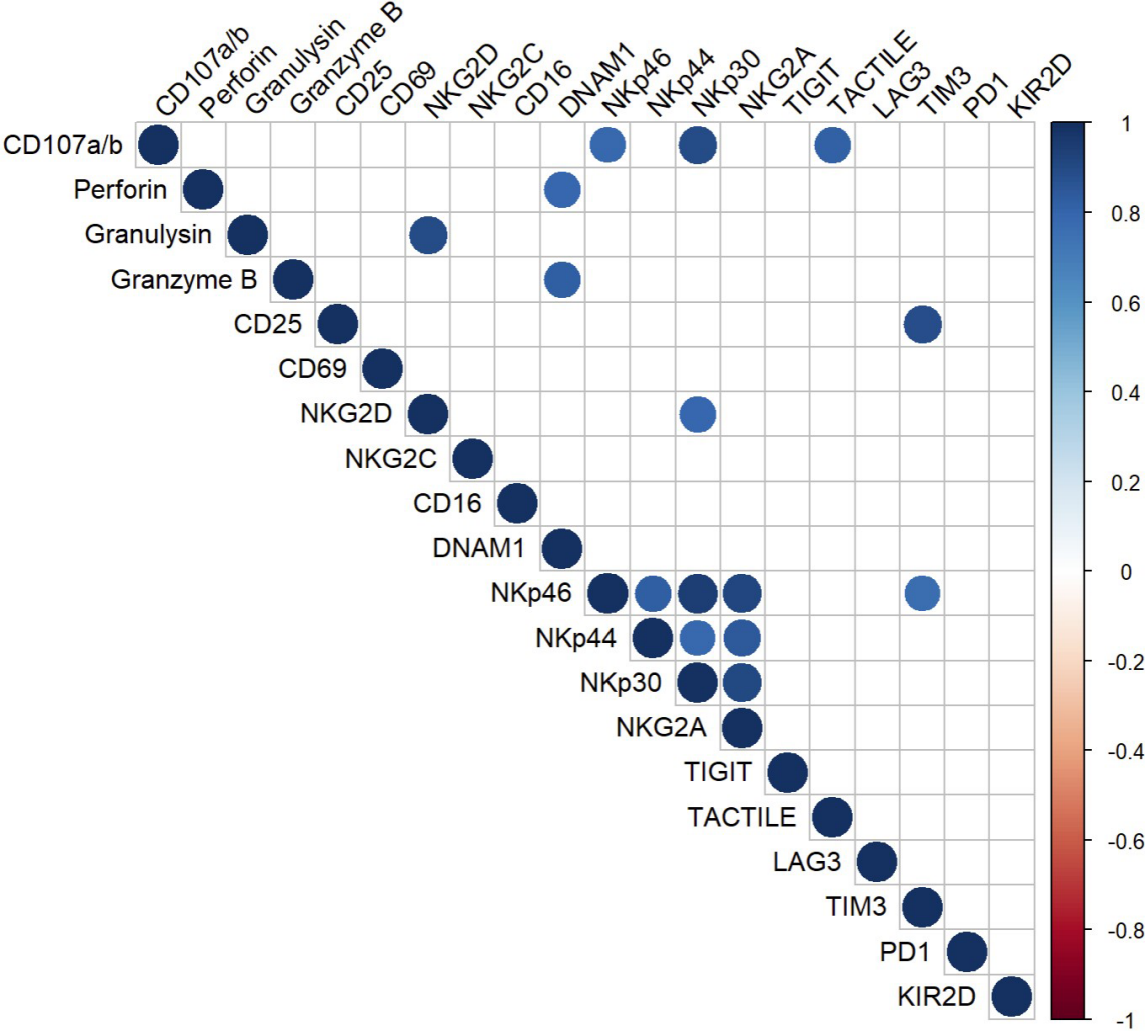
Multiple correlation analysis of CIML NK cells after 7 days of culture. The Pearson correlation coefficient was used to determine multiple correlations. The color and size of the circles in the graph correspond to the obtained *r-*value; only significant correlations (*p* ≤ 0.05) are displayed in the graph.

To establish a correlation between the functional and phenotypic profile of CIML NK cells a multiple correlation assay was conducted, analyzing the relationship between the degranulation capacity and the expression of activating and inhibitory receptors (**Figure 6**).

Positive correlations were observed between the degranulation capacity and the expression of the activating receptors NKp46 and NKp30, and the inhibitory receptor TACTILE. Moreover, the multiple correlation analysis revealed additional positive correlations between various surface markers and cytotoxic proteins. For instance, the activating receptor DNAM-1 showed a strong positive correlation with the cytotoxic proteins Granzyme B and Perforin; the activating receptor NKG2D, on the other hand, appeared to be strongly correlated with the cytotoxic protein Granulysin. Moreover, strong positive correlations were observed between the activating receptors NKG2D and NKp30, among the NCRs, and between the NCRs and the inhibitory receptor NKG2A.

The results from the multiple correlation analysis are represented in **Figure 6**. Only those correlations with *p* ≤ 0.05 are displayed. A full compilation of the *r*-values and *p*-values can be found in the **Supplementary Material** (**Supplementary Tables 4**, **5**, respectively).

## 4 Discussion

Natural killer cells play a critical role in innate immunity, as they are able to rapidly recognize and eliminate tumor-transformed and virus-infected cells without previous sensitization (1, 2, 13–16). In recent years, there has been an increasing interest in a distinct subset of NK cells with memory-like properties. Owing to their heightened effector function and longer half-life, CIML NK cells have become a promising tool in immunotherapy for various types of tumors (13, 20–24). In this study, we aimed to characterize the phenotypic and functional differences between *in vitro*-expanded CIML and control NK cells. Using multiparametric flow cytometry, we examined the impact of cytokine stimulation on NK cell functionality and surface marker expression, highlighting the distinct features of CIML NK cells and contributing to a deeper understanding of their biology and therapeutic potential.

Our phenotypic and functional studies provide compelling evidence for the distinguishable nature of CIML NK cells compared to control NK cells. After a short stimulation with IL-12, IL-15, and IL-18, surface marker analysis showed that CIML NK cells displayed a more activated phenotype than control NK cells, as evidenced by the increased expression of CD25 and CD69. As expected, these results are in agreement with previous reports showing the expression of these receptors in CIML NK cells (20, 24, 28, 29).

Phenotypic characterization of activating receptors revealed enhanced expression of NKG2D, NKp46, NKp44, and NKp30 in CIML NK cells after 7 days of culture, whereas the expression of other activating receptors (NKG2C, CD16, DNAM-1, and NKp80) was similar between CIML and control NK cells. Some of these results, such as the increase in NKG2D, NKG2C, and NCRs have previously been described in research articles and reviews (23, 29). Notably, both NKG2D and NCRs play crucial roles in NK cell effector responses by recognizing their ligands on tumor cells and mediating the production of cytokines and cytotoxic molecules (30, 31). Therefore, the upregulation of these activating receptors on CIML NK cells suggests an augmented capacity for target cell recognition and cytotoxicity in this subset of memory-like NK cells.

Moreover, in our comprehensive analysis of NK cell surface markers, we observed the presence of CD8 expression in about one third of both CIML and control NK cells. However, our statistical analysis did not reveal any statistically significant differences in CD8 expression between CIML and control NK cells after 7 days of culture. Notably, previous studies employing CIML NK cells in leukemia treatment have suggested a potential unfavorable connection between CD8 expression and clinical outcomes following *in vivo* adoptive cell transfer (32). Given these findings and the fact that the physiological role of CD8 on human NK cells is not yet fully understood, additional research is necessary to unveil the implications of CD8 expression and its potential impact on future immunotherapies (33).

In addition to activating receptors and CD8, the effect of cytokine stimulation on the expression profile of inhibitory receptors was also assessed, revealing heightened expression of NKG2A and TACTILE, and significant downregulation of KIR2D in CIML NK cells after 7 days of culture. However, the expression of TIGIT, LAG-3, TIM-3, and PD-1, was unaffected. Moreover, the upregulation of NKG2A (22, 23, 29) and TACTILE (34), as well as the downregulation of KIR2D (13, 35), have previously been observed by other authors. The upregulation of NKG2A and TACTILE observed in *in vitro*-expanded memory-like NK cells may negatively influence the functionality of adoptively transferred cells. This idea is supported by the fact that TACTILE binding to CD155 on human hepatocellular carcinoma cells contributes to immune escape by inducing NK cell exhaustion and reducing cytotoxicity and cytokine production (36). In contrast, the downregulation of KIR2D expression found in *in vitro*-expanded CIML NK cells may enhance their anti-tumor efficacy, as it has previously been demonstrated (35).

A comprehensive analysis of NK surface markers, such as the one presented in this study, could be of great value to identify more effective NK cell subsets prior to cell infusion in patients as suggested by Schwab et al. (37). Moreover, our phenotypic study has relevant and meaningful implications for identifying the surface markers that could be targeted in immunotherapeutic strategies, such as immune checkpoint inhibition. Many monoclonal antibodies (mAbs) are currently used to block the inhibitory pathways of NK cells and prevent immune evasion by allowing NK cells to efficiently recognize and eliminate tumor cells (38). The efficacy and safety of anti-NKG2A (monalizumab) and anti-KIR (lirilumab) mAbs have been demonstrated in various clinical trials (38–41), and they have shown promising results against hematological malignancies. TACTILE blockade has shown promising results in inhibiting metastatic progression in three different mouse tumor models (42) and could be a compelling candidate for future immunotherapies.

Following IL-12/15/18 stimulation, NK cells undergo a series of phenotypic changes characterized by the increased expression of CD69, NKG2D, NKG2A, and CD25 (14, 23). Our investigation also focused on the co-expression patterns of these markers, revealing a significantly higher abundance of the CD69+NKG2D+NKG2A+CD25+ subset in CIML NK cells than in control NK cells. In contrast, the CD69−NKG2D−NKG2A−CD25− subset was significantly more abundant in control NK cells than in CIML NK cells. Moreover, the co-expression analysis of NCRs revealed that the NKp46+NKp44+NKp30+ subset was significantly more abundant in CIML NK cells than in control NK cells. These findings demonstrate that CIML NK cells are defined by the co-expression of activating receptors, which is in agreement with previous studies describing the enhanced activation state of NK cells after cytokine stimulation (23). In addition, these marker combinations could potentially be useful in establishing guidelines for distinguishing memory from naïve NK cell populations.

Furthermore, analysis of the co-expression patterns of DNAM-1, TIGIT, and TACTILE revealed a decrease of DNAM-1−TIGIT+TACTILE+ subset in CIML NK cells compared to control NK cells. This phenotype is related to decreased effector functions and cytotoxicity of NK cells in AML patients (43). The lower abundance of this subset in CIML NK cells and the higher expression of the activating receptor DNAM-1 could suggest higher DNAM-1-mediated degranulation and cytotoxicity.

Notably, we observed a significant increase in the cytotoxic protein Granzyme B in CIML NK cells after 7 days of culture, while the percentage of Granulysin+ cells was similar between CIML and control NK cells. However, we observed a slight, albeit not significant, decrease in Perforin+ cells in CIML NK cells when compared to control NK cells, which has also been detected by other authors (44, 45) and could be explained by the inter-donor variability in Perforin expression observed in our samples. In addition, the higher expression of Granzyme B in CIML NK cells has previously been reported by other authors who have described increased lysis of leukemia target cells *in vitro* (23, 46).

Additionally, along with the phenotypic characterization of NK cell surface markers, we evaluated the degranulation capacity and intracellular cytokine production of CIML NK cells against K562, an NK-sensitive target cell line. Our findings are consistent with previous studies demonstrating that CIML NK cells exhibit enhanced degranulation capacity and higher IFN-γ production against the K562 cell line (23, 29, 47). Nonetheless, it is important to note that cytokine production displayed substantial donor-to-donor variability among the participants included in our study.

Finally, we performed a multiple correlation analysis to identify correlations between the phenotype and degranulation capacity of CIML NK cells. Our results revealed strong positive correlations between the degranulation capacity of CIML NK cells and the expression of a number of activating molecules (NKp46 and NKp30) and inhibitory receptors (TACTILE). These findings support the crucial role of NKp46, and NKp30 in NK cell function. Previous research has shown that antibody blockade of these receptors would result in a reduction of NK cell degranulation against target cells (48). Additionally, the expression of NCRs has been strongly associated with the ability of NK cells to degranulate and kill tumor cells (49, 50). Furthermore, although the exact function of TACTILE is not yet fully understood (12), this receptor is known to possess both activating and inhibitory motifs, suggesting its involvement in mediating positive and negative signals in NK cells (51). Moreover, it has been observed that TACTILE expression in NK cells directly inhibits IFN-γ production but does not affect NK cell degranulation (52). In addition, K562 cells express high levels of CD155 ligand (53), and it has been reported that TACTILE expression facilitates NK cell adhesion to CD155-expressing cells, thereby stimulating NK cell cytotoxicity (54).

Our study also identified positive correlations between activating receptors (NKG2D and DNAM-1) and cytotoxic proteins (Granulysin, Perforin, and Granzyme B). Both NKG2D and DNAM-1 are known to be involved in NK-cell mediated cytotoxicity by recognizing their ligands on target cells, triggering downstream signaling pathways that result in the degranulation of cytotoxic proteins (55–58).

Additionally, our multiple correlation analysis revealed strong positive correlations between NKG2D and NKp30 on CIML NK cells, as well as among the NCRs. The simultaneous expression of these receptors may synergistically contribute to tumor cell lysis (59–62). Furthermore, our findings also revealed positive correlations between the activating receptors CD25 and NKp46, and the inhibitory receptor TIM-3 in CIML NK cells. Although our understanding of the role of TIM-3 in NK cell biology remains limited, it is known that this inhibitory receptor is constitutively expressed on resting NK cells and can negatively impact NK cell cytotoxicity (63). In addition, TIM-3 has been associated with NK cell maturation and exhaustion, phenomena observed in human NK cells following continuous exposure to IL-15 or stimulation with IL-12/15/18 *in vitro* (64, 65).

Overall, our findings broaden the general understanding of *in vitro-*expanded CIML NK cells and their potential as antitumor effector cells. Our comprehensive characterization of the phenotype and functional profile of memory-like NK cells provides valuable insights into optimizing the expansion protocols of CIML NK cells and for the selection of potent NK cell subsets that could help improve the efficacy of immunotherapies. While further research is necessary to investigate the clinical applications of CIML NK cells and fully understand the underlying mechanisms driving their enhanced functionality, we believe that our biomarker panel holds significant potential for practical use in clinical settings and adoptive cell therapies.

## Data availability statement

The raw data supporting the conclusions of this article will be made available by the authors, without undue reservation.

## Ethics statement

The studies involving humans were approved by The ethics committee of the University of Extremadura (Ref.: 118//2020). The studies were conducted in accordance with the local legislation and institutional requirements. Written informed consent for participation was not required from the participants or the participants’ legal guardians/next of kin because blood samples were obtained from anonymous healthy adult donors under the supervision of the Blood Donation Center of Extremadura (Mérida, Spain).

## Author contributions

SC-S: Formal Analysis, Investigation, Methodology, Writing – review & editing, Conceptualization, Data curation, Writing – original draft. NL-S: Conceptualization, Formal Analysis, Investigation, Methodology, Writing – original draft, Writing – review & editing. MG-S: Conceptualization, Formal Analysis, Investigation, Methodology, Writing – original draft, Writing – review & editing. ES-H: Methodology, Writing – review & editing. AP: Formal Analysis, Writing – review & editing. FH: Methodology, Writing – review & editing. ED: Writing – review & editing, Supervision. RS: Writing – review & editing, Conceptualization, Funding acquisition. JC: Supervision, Writing – review & editing, Formal Analysis, Investigation, Methodology, Writing – original draft. RT: Conceptualization, Funding acquisition, Writing – review & editing, Data curation, Formal Analysis, Investigation, Methodology, Project administration, Resources, Supervision, Writing – original draft.
