# Supplementary material for: Enhanced expression of natural cytotoxicity receptors on cytokine-induced memory-like natural killer cells correlates with effector function
Source: Front Immunol. 2023 Oct 16;14:1256404. doi: 10.3389/fimmu.2023.1256404 (PMC10613704; doi:10.3389/fimmu.2023.1256404)
Supplement: Supplementary file 1 [file DataSheet_1.docx]

Supplementary Material

Enhanced expression of natural cytotoxicity receptors on cytokine-induced memory-like natural killer cells correlates with effector function

Sofía Carreira-Santos^1†^, Nelson López-Sejas^1†^, Marina González-Sánchez^1†^, Eva Sánchez-Hernández^1^, Alejandra Pera^2,3^, Fakhri Hassouneh^2^, Esther Durán^4^, Rafael Solana^2,3,5*‡^, Javier G. Casado^1,6,7,8*‡^, Raquel Tarazona^1,8 ‡^

^1^Immunology Unit, Department of Physiology, Universidad de Extremadura, Cáceres, Spain.

^2^Immunology and Allergy Group (GC01), Maimonides Biomedical Research Institute of Córdoba (IMIBIC), Córdoba, Spain.

^3^Department of Cell Biology, Physiology and Immunology, Universidad de Córdoba, Córdoba, Spain.

^4^Anatomy and Comparative Pathological Anatomy Unit. Department of Animal Medicine, Faculty of Veterinary Medicine, Universidad de Extremadura, Cáceres, Spain.

^5^Immunology and Allergy Service, Reina Sofia University Hospital, Cordoba, Spain.

^6^CIBER de Enfermedades Cardiovasculares, ISCIII, Madrid, Spain.

^7^RICORS-TERAV Network, ISCIII, Madrid, Spain.

^8^Institute of Molecular Pathology Biomarkers, University of Extremadura, Cáceres, Spain.

^†^These authors contributed equally to this work and share the first authorship.

^‡^These authors share senior authorship.

*** Correspondence:**Javier G. Casado jgarcas@unex.es
Rafael Solana rsolana@uco.es

# Supplementary Figures and Tables

## Supplementary Figures


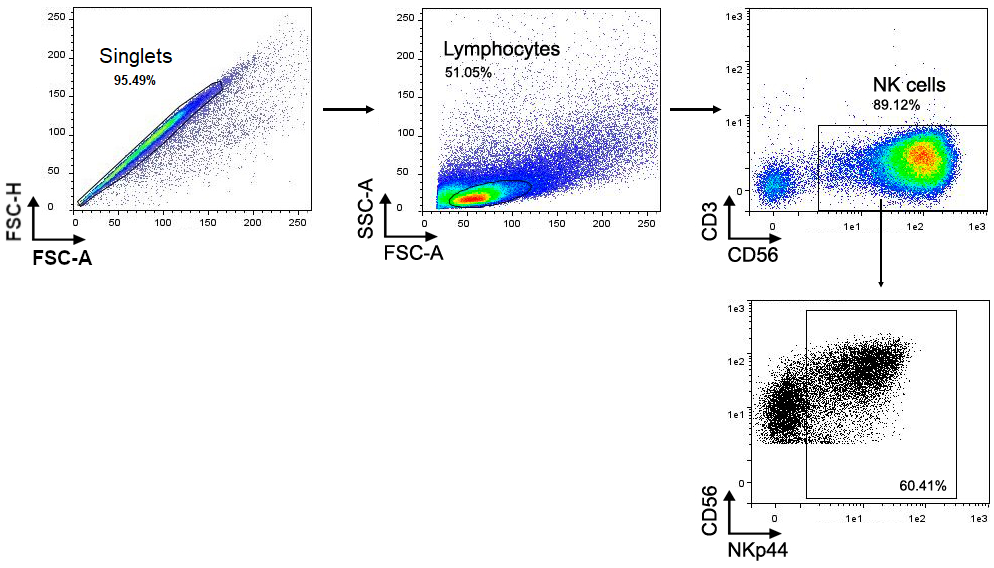


**Supplementary Figure 1.** **Gating strategy of CIML and control NK cells.** After doublet exclusion, Lymphocytes were gated according to their size and granularity using FSC and SSC detectors, and NK cells were identified within the lymphocyte gate as CD3− CD56+. Individual gates were defined for the rest of the antibodies included in the panel (e.g., NKp44) for the CD3− CD56+ cells.


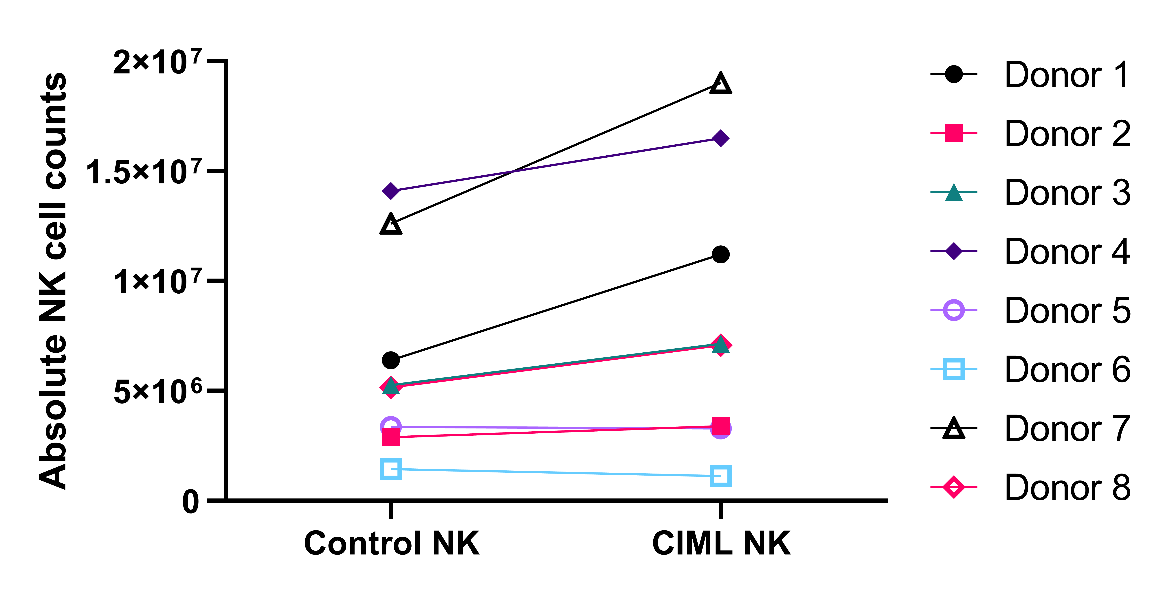


**Supplementary Figure 2.** **Representation of absolute cell counts after 7 days of culture of CIML and control NK cells.** On D7, absolute cell counts reveal that, in 6 out of the 8 donors included in this study, CIML NK cells displayed a higher absolute cell counts compared to control NK cells.


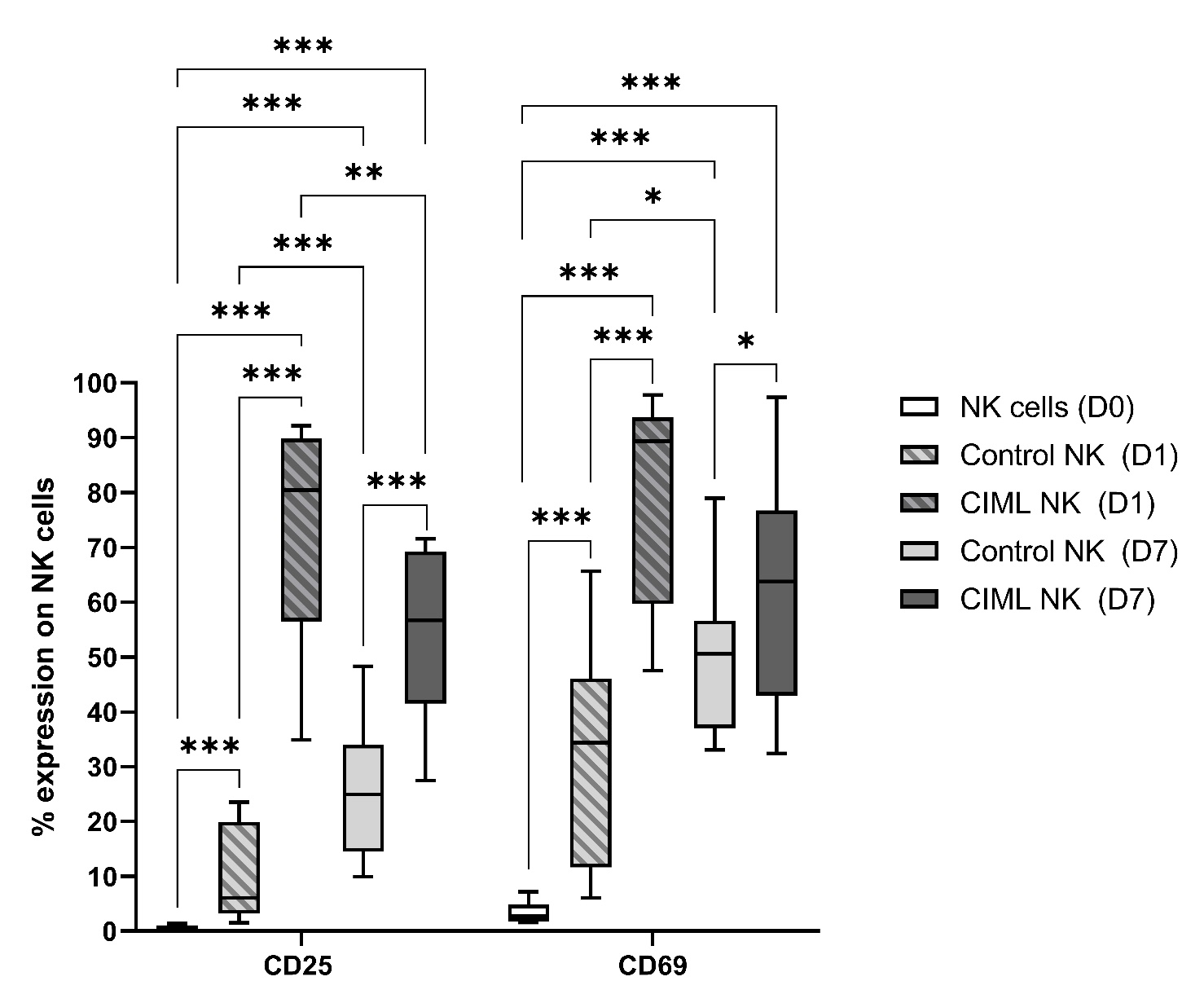


**Supplementary Figure 3. Evolution of NK cell activation markers in CIML and control NK cells over time (D0, D1, and D7).** On D0, NK cell expression of the activation markers CD25 and CD69 was relatively low, increasing significantly at D1 and D7 for both CIML and control NK cells (*p* < 0.001). On D1, CIML NK cells showed a significantly higher expression of both CD25 and CD69 than control NK cells (*p* < 0.001). On D7, CIML NK cells maintained a significantly higher expression of both CD25 (*p* < 0.001) and CD69 (*p* = 0.017) than control NK cells. Moreover, on D7 control NK cells showed a significant increase in both CD25 and CD69 when compared to D1 (*p* < 0.001 and *p* = 0,044, respectively). CIML NK cells, however, experimented significant downregulation of CD25 from D1 to D7 (*p* = 0,006). *p*-values were calculated by using the non-parametric Friedman test, followed by pairwise comparisons (Durbin-Conover test), * *p* ≤ 0.05, ** *p* ≤ 0.01, *** *p* ≤ 0.001.


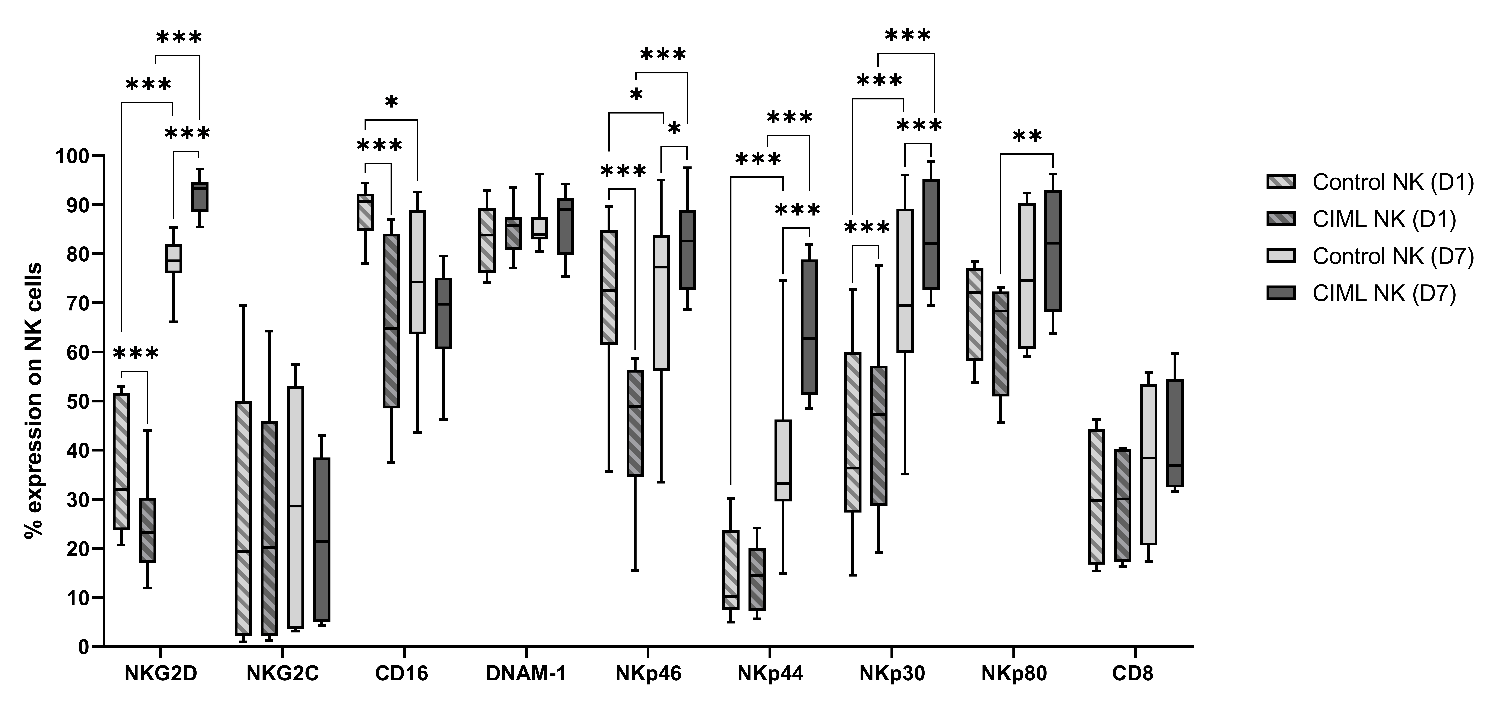


**Supplementary Figure 4. Evolution of NK cell activating receptors and CD8 expression in CIML and control NK cells over time (D1 and D7).** On D1, CIML NK cells exhibited significantly lower expression of NKG2D, CD16, and NKp46 than control NK cells (*p* < 0.001), contrary to NKp30 expression, which increased in CIML NK cells compared to control NK cells (*p* < 0.001). On D7, there was a significant upregulation of NKG2D, NKp44, NKp30 (*p* < 0.001, respectively), and NKp46 (*p* = 0,011) in CIML NK cells when compared to control NK cells. Both CIML and control NK cells showed significant upregulation of NKG2D, NKp44, NKp30 (*p* < 0.001, for both CIML and control NK cells), and NKp46 (*p* = 0,022 in control NK, *p* < 0.001 in CIML NK) from D1 to D7. Expression of NKp80 increased significantly from D1 to D7 in CIML NK cells (*p* = 0,002). *p*-values were calculated by using the non-parametric Friedman test, followed by pairwise comparisons (Durbin-Conover test), * *p* ≤ 0.05, ** *p* ≤ 0.01, *** *p* ≤ 0.001.


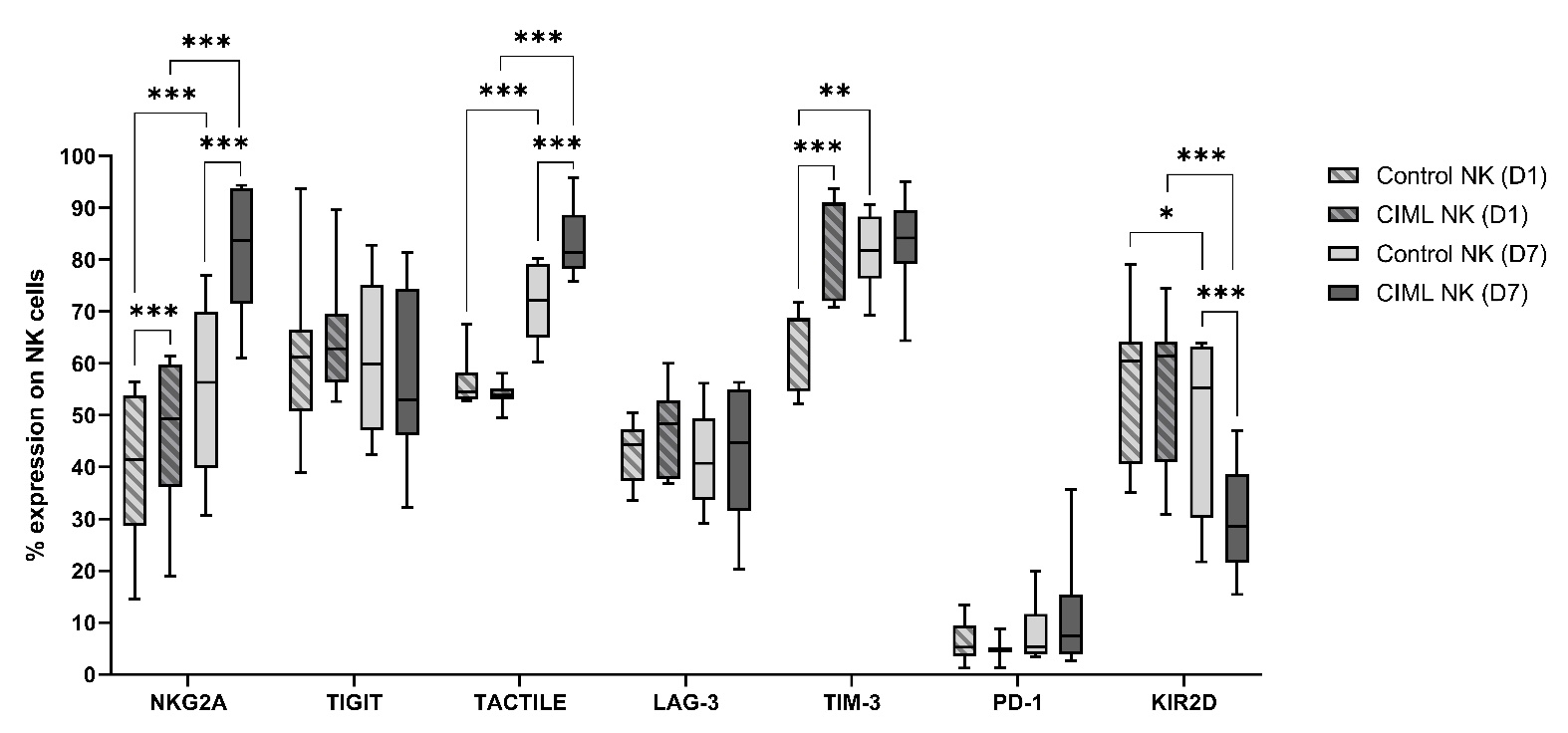


**Supplementary Figure 5. Evolution of NK cell inhibitory receptors in CIML and control cells over time (D1 and D7).** On D1, CIML NK cells expressed significantly higher percentages of NKG2A and TIM-3 than control NK cells (*p* < 0.001). On D7, CIML NK cells showed an increased expression of NKG2A and TACTILE (*p* < 0.001) than control NK cells; KIR2D expression, however, was significantly lower in CIML NK cells than in control NK cells (*p* < 0.001). Both CIML and control NK cells showed a significant increase in the expression of NKG2A and TACTILE (*p* < 0.001, respectively) from D1 to D7, while TIM-3 levels were significantly higher on D7 than on D1 in control NK cells (*p* = 0.003). Moreover, KIR2D expression experienced a significant decrease from D1 to D7 in both control *p* = 0.015) and CIML NK cells (*p* < 0.001). *p*-values were calculated by using the non-parametric Friedman test, followed by pairwise comparisons (Durbin-Conover test), * *p* ≤ 0.05, ** *p* ≤ 0.01, *** *p* ≤ 0.001.


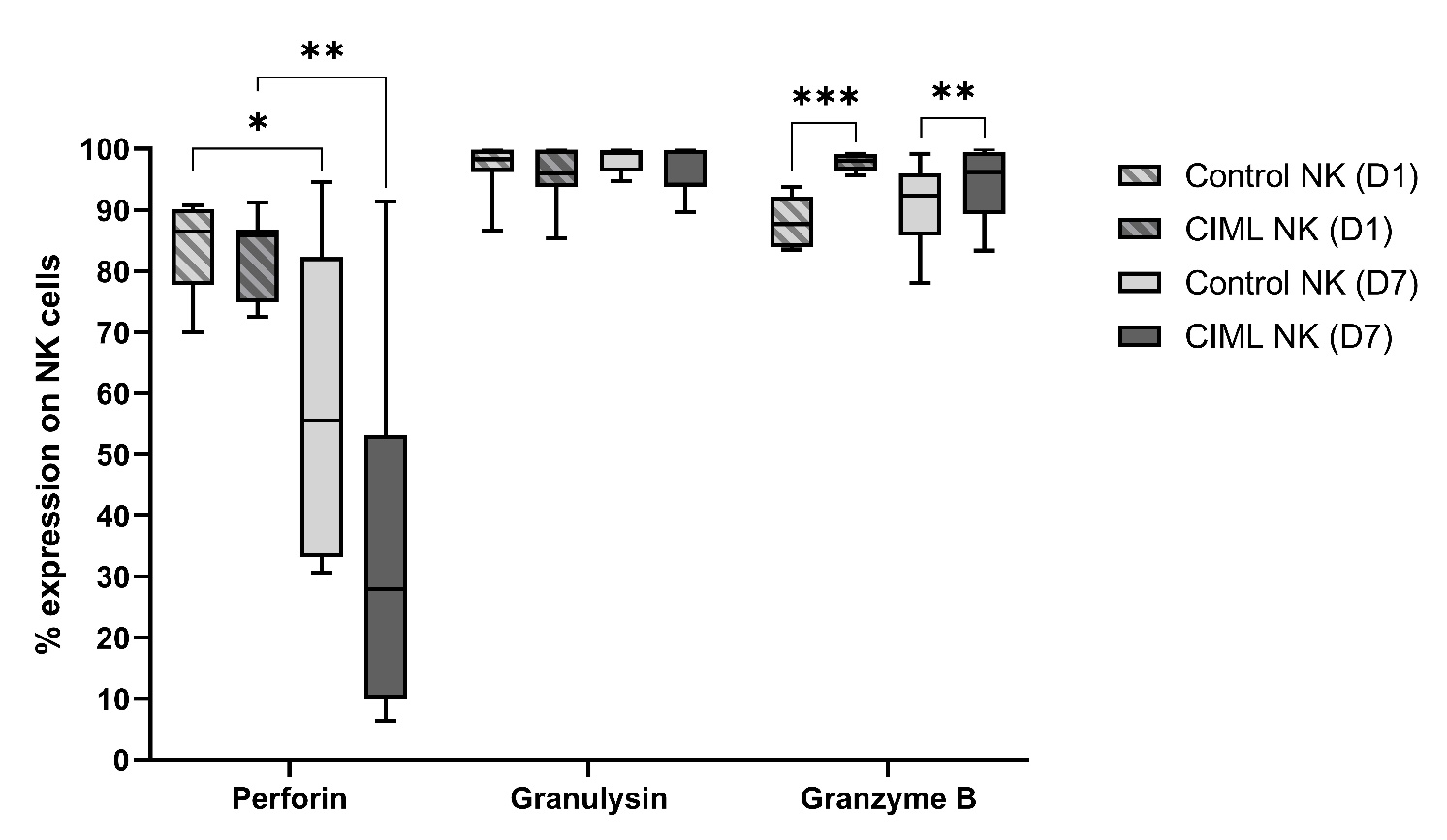


**Supplementary Figure 6. Evolution of NK cell cytotoxic proteins in CIML and control NK cells over time (D1 and D7).** Both on D1 and D7, CIML NK cells showed significantly higher expression of Granzyme B than control NK cells (*p* < 0.001 for D1, *p* = 0.005 for D7). Moreover, the expression of Perforin decreases significantly from D1 to D7 in both CIML and control NK cells (*p* = 0.017 and *p* = 0.002, respectively). *p*-values were calculated by using the non-parametric Friedman test, followed by pairwise comparisons (Durbin-Conover test), * *p* ≤ 0.05, ** *p* ≤ 0.01, *** *p* ≤ 0.001.


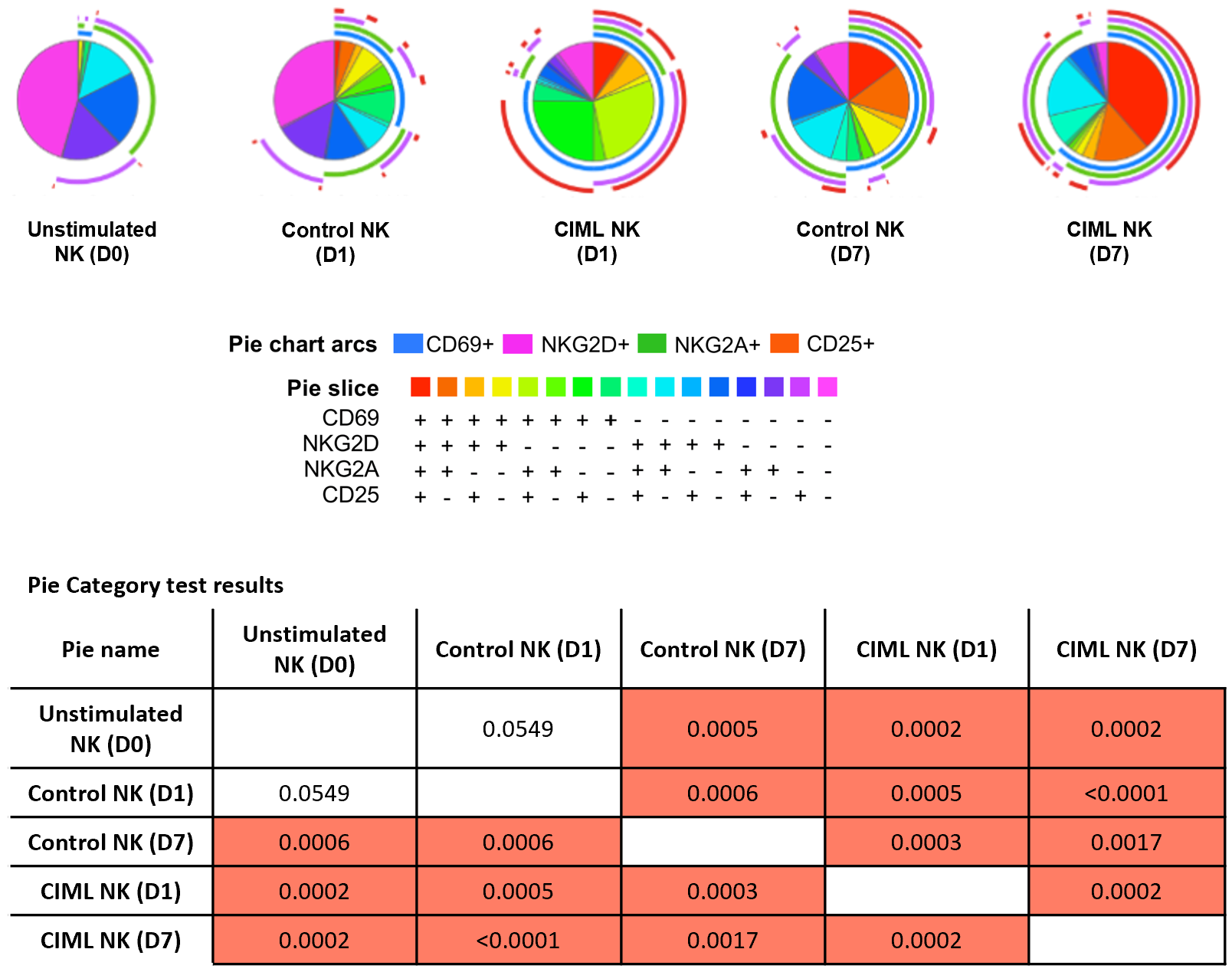


**Supplementary Figure 7. Co-expression of activating and inhibitory receptors in CIML and control NK cells at different time points (D0, D1, and D7).** Pie charts represent the percentages of CIML and control NK cells expressing CD69, NKG2D, NKG2A, and CD25. The *p*-values obtained in the pie category test analysis are compiled in the table shown above and were calculated by a non-parametric partial permutation test.

## Supplementary Tables

**Supplementary Table 1. Antibody panel comprising the extracellular antibodies used for the phenotypic characterization of NK cells.**

|  | Antibody | Fluorochrome | Provider | Clone |
| --- | --- | --- | --- | --- |
| tube 1 | CD16 | VioBlue® | Miltenyi Biotec | REA423 |
|  | CD3 | VioGreen® | Miltenyi Biotec | REA613 |
|  | CD25 | FITC | Invitrogen | CD25-3G10 |
|  | NKG2A | PE-REA | Miltenyi Biotec | REA110 |
|  | CD8 | PerCP-Cy5.5 | BD Biosciences | SK1 |
|  | CD56 | PE-Vio®770 | Miltenyi Biotec | REA196 |
|  | NKG2D | APC | BD Biosciences | 1D11 |
|  | CD69 | APC-Vio®770 | Miltenyi Biotec | REA824 |
| tUBE 2 | CD16 | VioBlue® | Miltenyi Biotec | REA423 |
|  | CD3 | VioGreen® | Miltenyi Biotec | REA613 |
|  | DNAM-1 | FITC | BD Biosciences | DX11 |
|  | TIGIT | PE | Invitrogen | MBSA43 |
|  | CD8 | PerCP-Cy5.5 | BD Biosciences | SK1 |
|  | CD56 | PE-Vio®770 | Miltenyi Biotec | REA196 |
|  | TACTILE | APC-REA | Miltenyi Biotec | REA195 |
|  | NKp80 | APC-Vio®770 | Miltenyi Biotec | REA845 |
| tube 3 | Lag-3 | BV421 | BD Biosciences | T47-530 |
|  | CD3 | VioGreen® | Miltenyi Biotec | REA613 |
|  | TIM-3 | BB515 | BD Biosciences | 7D3 |
|  | CD8 | PerCP-Cy5.5 | BD Biosciences | SK1 |
|  | CD56 | PE-Vio®770 | Miltenyi Biotec | REA196 |
|  | PD-1 | APC | BD Biosciences | MIH4 |
|  | CD16 | APC-Vio®770 | Miltenyi Biotec | REA423 |
| TUBE 4 | CD16 | VioBlue® | Miltenyi Biotec | REA423 |
|  | CD3 | VioGreen® | Miltenyi Biotec | REA613 |
|  | NKp44 | Vio®Bright-FITC | Miltenyi Biotec | 2.29 |
|  | NKp46 | PE | BD Biosciences | 9-E2 |
|  | CD8 | PerCP-Cy5.5 | BD Biosciences | SK1 |
|  | CD56 | PE-Vio®770 | Miltenyi Biotec | REA196 |
|  | NKp30 | APC | Miltenyi Biotec | AF29-4D12 |
|  | NKp80 | APC-Vio®770 | Miltenyi Biotec | REA845 |
| tUBE 5 | CD16 | VioBlue® | Miltenyi Biotec | REA423 |
|  | CD3 | VioGreen® | Miltenyi Biotec | REA613 |
|  | NKG2C | FITC | Miltenyi Biotec | REA205 |
|  | KIR2D | PE-REA | Miltenyi Biotec | REA1042 |
|  | CD8 | PerCP-Cy5.5 | BD Biosciences | SK1 |
|  | CD56 | PE-Vio®770 | Miltenyi Biotec | REA196 |
|  | NKp80 | APC-Vio®770 | Miltenyi Biotec | REA845 |

**Supplementary Table 2. Antibody panel comprising the intracellular antibodies used for the phenotypic characterization of NK cells.**

|  | Antibody | Fluorochrome | Provider | Clone |
| --- | --- | --- | --- | --- |
| INTRACELLULAR TUBE | Perforin | VioBlue® | Miltenyi Biotec | 𝛿G9 |
|  | CD3 | VioGreen® | Miltenyi Biotec | REA613 |
|  | Granulysin | AF488 | BD Biosciences | RB1 |
|  | CD16 | PE-REA | Miltenyi Biotec | REA423 |
|  | CD8 | PerCP-Cy5.5 | BD Biosciences | SK1 |
|  | CD56 | PE-Vio®770 | Miltenyi Biotec | REA196 |
|  | Granzyme B | AF647 | BD Biosciences | GB11 |
|  | NKp80 | APC-Vio®770 | Miltenyi Biotec | REA845 |

**Supplementary Table 3. Antibody panel comprising the extracellular and intracellular antibodies used for the functional analysis of NK cells.**

| Extracellular staining | | | |
| --- | --- | --- | --- |
| Antibody | **Fluorochrome** | **Provider** | **Clone** |
| CD107a | FITC | BD Biosciences | H4A3 |
| CD107b | FITC | BD Biosciences | H4B4 |
| CD16 | VioBlue® | Miltenyi Biotec | REA423 |
| CD3 | VioGreen® | Miltenyi Biotec | REA613 |
| CD56 | PE-Vio®770 | Miltenyi Biotec | REA196 |
| CD8 | PerCP-Cy5.5 | BD Biosciences | SK1 |
| NKp80 | APC-Vio®770 | Miltenyi Biotec | REA845 |
| Intracellular staining | | | |
| Antibody | **Fluorochrome** | **Provider** | **Clone** |
| ifn-γ | APC | BD Biosciences | B27 |
| tnf-α | PE | R&D Systems | 6402 |

**Supplementary Table 4. Compilation of the *r*-values from the correlation matrix obtained by the Pearson correlation coefficient for CIML NK cells.**

**CD107a/b Perforin Granulysin Granzyme B**

**CD107a/b** 1.00 0.69 0.46 0.64

**Perforin** 0.69 1.00 -0.18 0.46

**Granulysin** 0.46 -0.18 1.00 0.31

**Granzyme B** 0.64 0.46 0.31 1.00

**CD25** 0.02 -0.06 0.33 -0.60

**CD69** 0.40 0.57 -0.16 -0.14

**NKG2D** 0.75 0.25 0.89 0.58

**NKG2C** 0.06 -0.08 0.19 0.52

**CD16** 0.20 0.31 -0.17 0.35

**DNAM1** 0.75 0.80 -0.05 0.82

**NKp46** 0.78 0.40 0.49 0.16

**NKp44** 0.59 0.17 0.27 -0.02

**NKp30** 0.89 0.39 0.64 0.36

**NKG2A** 0.72 0.25 0.40 0.11

**TIGIT** 0.20 0.29 0.31 0.15

**TACTILE** 0.82 0.71 0.38 0.30

**LAG3** -0.28 -0.28 -0.12 -0.18

**TIM3** 0.42 0.36 0.35 -0.23

**PD1** 0.70 0.75 0.11 0.45

**KIR2D** 0.09 0.20 0.42 0.43

**CD25 CD69 NKG2D NKG2C CD16 DNAM1 NKp46 NKp44 NKp30**

**CD107a/b** 0.02 0.40 0.75 0.06 0.20 0.75 0.78 0.59 0.89

**Perforin** -0.06 0.57 0.25 -0.08 0.31 0.80 0.40 0.17 0.39

**Granulysin** 0.33 -0.16 0.89 0.19 -0.17 -0.05 0.49 0.27 0.64

**Granzyme B** -0.60 -0.14 0.58 0.52 0.35 0.82 0.16 -0.02 0.36

**CD25**  1.00 0.54 0.23 -0.24 -0.29 -0.52 0.52 0.39 0.33

**CD69**  0.54 1.00 0.06 0.09 0.36 0.17 0.61 0.64 0.42

**NKG2D**  0.23 0.06 1.00 0.24 -0.09 0.36 0.67 0.32 0.80

**NKG2C**  -0.24 0.09 0.24 1.00 0.50 0.16 -0.02 0.07 0.03

**CD16**  -0.29 0.36 -0.09 0.50 1.00 0.24 -0.13 0.18 -0.04

**DNAM1**  -0.52 0.17 0.36 0.16 0.24 1.00 0.33 0.13 0.43

**NKp46** 0.52 0.61 0.67 -0.02 -0.13 0.33 1.00 0.82 0.95

**NKp44** 0.39 0.64 0.32 0.07 0.18 0.13 0.82 1.00 0.78

**NKp30** 0.33 0.42 0.80 0.03 -0.04 0.43 0.95 0.78 1.00

**NKG2A** 0.31 0.39 0.52 -0.18 -0.24 0.33 0.91 0.85 0.90

**TIGIT**  0.40 0.47 0.35 0.41 0.58 -0.06 0.12 0.05 0.12

**TACTILE**  0.25 0.32 0.61 -0.43 -0.04 0.52 0.63 0.32 0.70

**LAG3** 0.24 0.43 -0.18 0.71 0.31 -0.31 0.05 0.29 -0.10

**TIM3** 0.88 0.75 0.46 -0.12 -0.13 -0.07 0.76 0.53 0.60

**PD1**  0.10 0.71 0.38 0.31 0.74 0.53 0.46 0.45 0.49

**KIR2D**  0.02 -0.22 0.50 0.26 0.02 0.13 -0.15 -0.57 -0.07

**NKG2A TIGIT TACTILE LAG3 TIM3 PD1 KIR2D**

**CD107a/b** 0.72 0.20 0.82 -0.28 0.42 0.70 0.09

**Perforin** 0.25 0.29 0.71 -0.28 0.36 0.75 0.20

**Granulysin** 0.40 0.31 0.38 -0.12 0.35 0.11 0.42

**Granzyme B** 0.11 0.15 0.30 -0.18 -0.23 0.45 0.43

**CD25** 0.31 0.40 0.25 0.24 0.88 0.10 0.02

**CD69** 0.39 0.47 0.32 0.43 0.75 0.71 -0.22

**NKG2D** 0.52 0.35 0.61 -0.18 0.46 0.38 0.50

**NKG2C** -0.18 0.41 -0.43 0.71 -0.12 0.31 0.26

**CD16** -0.24 0.58 -0.04 0.31 -0.13 0.74 0.02

**DNAM1** 0.33 -0.06 0.52 -0.31 -0.07 0.53 0.13

**NKp46** 0.91 0.12 0.63 0.05 0.76 0.46 -0.15

**NKp44** 0.85 0.05 0.32 0.29 0.53 0.45 -0.57

**NKp30** 0.90 0.12 0.70 -0.10 0.60 0.49 -0.07

**NKG2A** 1.00 -0.24 0.55 -0.09 0.50 0.23 -0.43

**TIGIT** -0.24 1.00 0.21 0.30 0.49 0.70 0.59

**TACTILE** 0.55 0.21 1.00 -0.63 0.54 0.54 0.23

**LAG3** -0.09 0.30 -0.63 1.00 0.17 0.12 -0.19

**TIM3** 0.50 0.49 0.54 0.17 1.00 0.46 0.12

**PD1** 0.23 0.70 0.54 0.12 0.46 1.00 0.18

**KIR2D** -0.43 0.59 0.23 -0.19 0.12 0.18 1.00

**Supplementary Table 5. Compilation of the *p*-values from the correlation matrix obtained by the Pearson correlation coefficient for CIML NK cells.** Significant values are highlighted in red.

**CD107a/b Perforin Granulysin Granzyme B**

**CD107a/b** 0.0831 0.3041 0.1242

**Perforin** 0.0831 0.7074 0.2958

**Granulysin** 0.3041 0.7074 0.4924

**Granzyme B** 0.1242 0.2958 0.4924

**CD25** 0.9679 0.8936 0.4768 0.1572

**CD69** 0.3752 0.1827 0.7303 0.7680

**NKG2D** 0.0540 0.5837 0.0072 0.1715

**NKG2C** 0.9025 0.8583 0.6819 0.2308

**CD16** 0.6597 0.4985 0.7235 0.4482

**DNAM1** 0.0540 0.0323 0.9185 0.0236

**NKp46** 0.0382 0.3706 0.2649 0.7390

**NKp44** 0.1669 0.7212 0.5620 0.9583

**NKp30** 0.0071 0.3895 0.1244 0.4215

**NKG2A** 0.0675 0.5872 0.3778 0.8126

**TIGIT** 0.6654 0.5297 0.5053 0.7549

**TACTILE** 0.0247 0.0743 0.4018 0.5112

**LAG3** 0.5374 0.5408 0.7986 0.7019

**TIM3** 0.3477 0.4210 0.4456 0.6131

**PD1** 0.0804 0.0521 0.8174 0.3138

**KIR2D** 0.8447 0.6675 0.3451 0.3335

**CD25 CD69 NKG2D NKG2C CD16 DNAM1 NKp46 NKp44 NKp30**

**CD107a/b** 0.9679 0.3752 0.0540 0.9025 0.6597 0.0540 0.0382 0.1669 0.0071

**Perforin** 0.8936 0.1827 0.5837 0.8583 0.4985 0.0323 0.3706 0.7212 0.3895

**Granulysin** 0.4768 0.7303 0.0072 0.6819 0.7235 0.9185 0.2649 0.5620 0.1244

**Granzyme B** 0.1572 0.7680 0.1715 0.2308 0.4482 0.0236 0.7390 0.9583 0.4215

**CD25** 0.2145 0.6249 0.6060 0.5340 0.2334 0.2353 0.3923 0.4751

**CD69** 0.2145 0.8951 0.8415 0.4304 0.7190 0.1478 0.1201 0.3442

**NKG2D** 0.6249 0.8951 0.5986 0.8533 0.4226 0.1002 0.4845 0.0320

**NKG2C** 0.6060 0.8415 0.5986 0.2536 0.7332 0.9738 0.8793 0.9452

**CD16** 0.5340 0.4304 0.8533 0.2536 0.5974 0.7738 0.6925 0.9387

**DNAM1** 0.2334 0.7190 0.4226 0.7332 0.5974 0.4753 0.7782 0.3340

**NKp46** 0.2353 0.1478 0.1002 0.9738 0.7738 0.4753 0.0237 0.0011

**NKp44** 0.3923 0.1201 0.4845 0.8793 0.6925 0.7782 0.0237 0.0372

**NKp30** 0.4751 0.3442 0.0320 0.9452 0.9387 0.3340 0.0011 0.0372

**NKG2A** 0.4997 0.3823 0.2336 0.7040 0.6100 0.4735 0.0040 0.0161 0.0051

**TIGIT** 0.3802 0.2894 0.4448 0.3595 0.1679 0.8935 0.7975 0.9124 0.7944

**TACTILE** 0.5940 0.4890 0.1458 0.3310 0.9298 0.2338 0.1283 0.4871 0.0801

**LAG3** 0.6044 0.3309 0.7067 0.0739 0.4985 0.4920 0.9081 0.5220 0.8314

**TIM3** 0.0081 0.0514 0.3024 0.7905 0.7844 0.8862 0.0454 0.2251 0.1552

**PD1** 0.8268 0.0711 0.4020 0.5024 0.0596 0.2259 0.3009 0.3050 0.2597

**KIR2D** 0.9585 0.6419 0.2533 0.5700 0.9669 0.7873 0.7426 0.1849 0.8824

**NKG2A TIGIT TACTILE LAG3 TIM3 PD1 KIR2D**

**CD107a/b** 0.0675 0.6654 0.0247 0.5374 0.3477 0.0804 0.8447

**Perforin** 0.5872 0.5297 0.0743 0.5408 0.4210 0.0521 0.6675

**Granulysin** 0.3778 0.5053 0.4018 0.7986 0.4456 0.8174 0.3451

**Granzyme B** 0.8126 0.7549 0.5112 0.7019 0.6131 0.3138 0.3335

**CD25** 0.4997 0.3802 0.5940 0.6044 0.0081 0.8268 0.9585

**CD69** 0.3823 0.2894 0.4890 0.3309 0.0514 0.0711 0.6419

**NKG2D** 0.2336 0.4448 0.1458 0.7067 0.3024 0.4020 0.2533

**NKG2C** 0.7040 0.3595 0.3310 0.0739 0.7905 0.5024 0.5700

**CD16** 0.6100 0.1679 0.9298 0.4985 0.7844 0.0596 0.9669

**DNAM1** 0.4735 0.8935 0.2338 0.4920 0.8862 0.2259 0.7873

**NKp46** 0.0040 0.7975 0.1283 0.9081 0.0454 0.3009 0.7426

**NKp44** 0.0161 0.9124 0.4871 0.5220 0.2251 0.3050 0.1849

**NKp30** 0.0051 0.7944 0.0801 0.8314 0.1552 0.2597 0.8824

**NKG2A** 0.6037 0.1967 0.8407 0.2517 0.6146 0.3300

**TIGIT** 0.6037 0.6510 0.5063 0.2589 0.0781 0.1655

**TACTILE** 0.1967 0.6510 0.1272 0.2123 0.2150 0.6273

**LAG3**  0.8407 0.5063 0.1272 0.7139 0.7928 0.6809

**TIM3** 0.2517 0.2589 0.2123 0.7139 0.3044 0.8001

**PD1** 0.6146 0.0781 0.2150 0.7928 0.3044 0.6947

**KIR2D** 0.3302 0.1655 0.6273 0.6809 0.8001 0.6947
